# Three-dimensional organotypic co-culture model of intestinal epithelial cells and macrophages to study *Salmonella enterica* colonization patterns

**DOI:** 10.1038/s41526-017-0011-2

**Published:** 2017-02-28

**Authors:** Jennifer Barrila, Jiseon Yang, Aurélie Crabbé, Shameema F. Sarker, Yulong Liu, C. Mark Ott, Mayra A. Nelman-Gonzalez, Simon J. Clemett, Seth D. Nydam, Rebecca J. Forsyth, Richard R. Davis, Brian E. Crucian, Heather Quiriarte, Kenneth L. Roland, Karen Brenneman, Clarence Sams, Christine Loscher, Cheryl A. Nickerson

**Affiliations:** 1 Center for Infectious Diseases and Vaccinology, The Biodesign Institute, Arizona State University, Tempe, AZ USA; 2 Laboratory of Pharmaceutical Microbiology, Ghent University, Ghent, Belgium; 3 Biomedical Research and Environmental Sciences Division, NASA Johnson Space Center, Houston, TX USA; 4 Wyle Science, Technology and Engineering Group, Houston, TX USA; 5 ERC, Inc/Jacobs JETS, Houston, TX USA; 6 JES Tech, Houston, TX USA; 7 Immunomodulation Research Group, School of Biotechnology, Dublin City University, Glasnevin, Ireland; 8 School of Life Sciences, Arizona State University, Tempe, AZ USA

## Abstract

Three-dimensional models of human intestinal epithelium mimic the differentiated form and function of parental tissues often not exhibited by two-dimensional monolayers and respond to *Salmonella* in key ways that reflect in vivo infections. To further enhance the physiological relevance of three-dimensional models to more closely approximate in vivo intestinal microenvironments encountered by *Salmonella*, we developed and validated a novel three-dimensional co-culture infection model of colonic epithelial cells and macrophages using the NASA Rotating Wall Vessel bioreactor. First, U937 cells were activated upon collagen-coated scaffolds. HT-29 epithelial cells were then added and the three-dimensional model was cultured in the bioreactor until optimal differentiation was reached, as assessed by immunohistochemical profiling and bead uptake assays. The new co-culture model exhibited in vivo-like structural and phenotypic characteristics, including three-dimensional architecture, apical-basolateral polarity, well-formed tight/adherens junctions, mucin, multiple epithelial cell types, and functional macrophages. Phagocytic activity of macrophages was confirmed by uptake of inert, bacteria-sized beads. Contribution of macrophages to infection was assessed by colonization studies of *Salmonella* pathovars with different host adaptations and disease phenotypes (Typhimurium ST19 strain SL1344 and ST313 strain D23580; Typhi Ty2). ﻿﻿In addition, *Salmonella* were cultured aerobically or microaerobically, recapitulating environments encountered prior to and during intestinal infection, respectively﻿. All *Salmonella* strains exhibited decreased colonization in co-culture (HT-29-U937) relative to epithelial (HT-29) models, indicating antimicrobial function of macrophages. Interestingly, D23580 exhibited enhanced replication/survival in both models following invasion. Pathovar-specific differences in colonization and intracellular co-localization patterns were observed. These findings emphasize the power of incorporating a series of related three-dimensional models within a study to identify microenvironmental factors important for regulating infection.

## Modeling intestinal infection with NASA biotechnology: A new 3-D intestinal co-culture model with macrophages to study enteric infection

Using spaceflight analog bioreactor technology, Cheryl Nickerson at Arizona State University and collaborators developed and validated a new three-dimensional (3-D) intestinal co-culture model containing multiple differentiated epithelial cell types and phagocytic macrophages with antibacterial function to study infection by multiple pathovars of *Salmonella.* This study is the first to show that these pathovars (known to possess different host adaptations, antibiotic resistance profiles and disease phenotypes)*,* display markedly different colonization and intracellular co-localization patterns using this physiologically relevant new 3-D intestinal co-culture model. This advanced model, that integrates a key immune cell type important for *Salmonella* infection, offers a powerful new tool in understanding enteric pathogenesis and may lead to unexpected pathogenesis mechanisms and therapeutic targets that have been previously unobserved or unappreciated using other intestinal cell culture models.

## Introduction

In vitro cell culture models comprised solely of either epithelial cells or macrophages cultured as monolayers are often used to study *Salmonella enterica* infections, as both cell types play critical roles in the infection process.^1, 2^ Following ingestion, *Salmonella* actively invade and replicate within intestinal epithelial cells and are engulfed by macrophages upon crossing the epithelial barrier, where they exploit phagocytes as a preferred niche for replication and transport.^3^ While classic two-dimensional (2-D) monolayers composed of a single cell type have provided important insight into understanding the interactions between *Salmonella* and host tissues during enteric infection, they lack the multicellular complexity and three-dimensional (3-D) architecture that are important for the differentiated structure and function of the in vivo parental tissue.^4–7^ Synergism between epithelial cells and macrophages is important for driving responses of both cell types.^5, 8, 9^ Not surprisingly, monotypic (single cell type) cultures of either epithelial cells or macrophages respond differently to challenge with pathogens or their toxins as compared to co-culture models containing both of these cell types, the latter of which demonstrate a synergistic phenotype that better reflect the in vivo response.^5, 10^ Indeed, it is widely recognized there is an urgent need for advanced in vitro cell culture models that mimic the complex 3-D architecture, multicellular complexity and phenotypic characteristics of in vivo tissues for use in predictive human disease modeling, including infectious disease.^4, 5, 11–13^ Accordingly, achieving a deeper understanding of host–pathogen interactions at the intestinal mucosa requires cell culture models that incorporate 3-D architecture, differentiation and multicellular complexity to characterize the interaction between epithelial cells and macrophages during enteric infection.

**Fig. 1:**
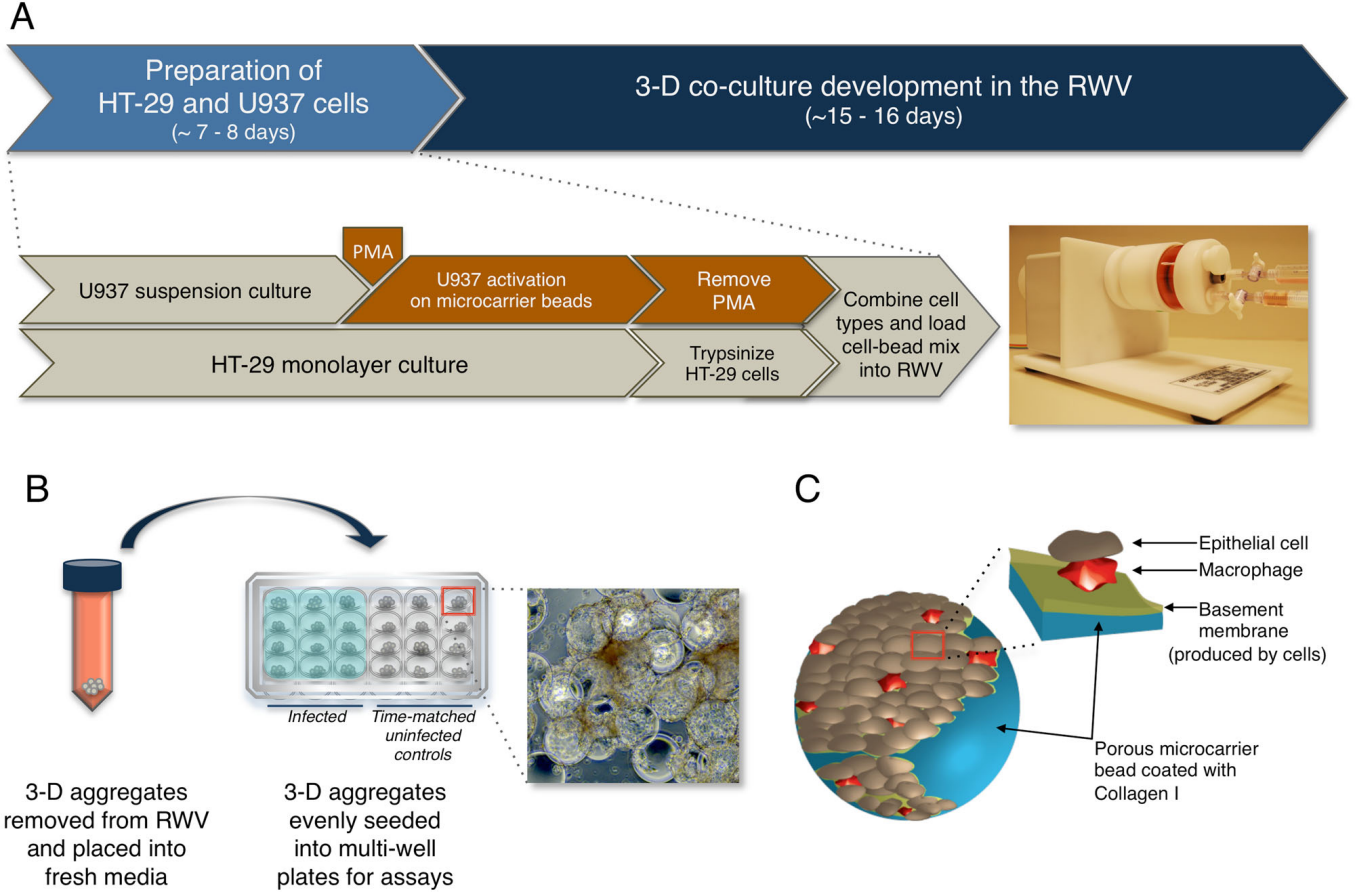
Development strategy for 3-D co-culture model of human intestinal epithelium and macrophages. **A** Experimental design for 3-D co-culture model development. HT-29 cells were initiated as adherent monolayers and U937 cells as suspension cultures. U937 cells (1 × 10^7^) were treated with PMA for 48 h in the presence of ECM-coated, porous microcarrier beads to allow attachment. Following treatment, PMA was removed from the U937 cells. At the same time, HT-29 cells were trypsinized, counted and added (2 × 10^6^) to the U937-bead mix. The RWV was loaded with cultures, filled completely with media, and bubbles removed. Routine daily media changes began 4–5 days after initiation of cultures. **B** Assay set-up for infection studies. 3-D aggregates were removed from the RWV, counted and evenly seeded into multi-well plates. Light micrograph magnification = 100×. **C** Depiction of a single collagen-coated microcarrier bead (*blue*) covered with HT-29 cells (*brown*) and U937 cells (*red*)

The rotating wall vessel (RWV) bioreactor is a NASA biotechnology that was designed to mimic aspects of the quiescent, low fluid shear culture environment found in microgravity in order to facilitate the formation of 3-D tissue-like aggregates in the laboratory.^4, 14, 15^ This low fluid shear suspension culture system has served as a powerful ground-based tool to enable the establishment of highly differentiated 3-D cell cultures, including both mono﻿typic (originating from a single cell﻿ type) and co-cultures (multiple cell types)^4, 10^ and has been used to establish highly differentiated 3-D intestinal epithelial models to study enteric infections.^4, 7, 16–20^ Our lab was first to apply RWV-derived 3-D cell cultures to study bacterial host–pathogen interactions, including 3-D intestinal epithelial models for *Salmonella* pathogenesis,^7, 17, 18^ and 3-D lung epithelial models co-cultured with macrophages for *Pseudomonas aeruginosa* pathogenesis.^10^ The underlying concept of the RWV (Fig. 1) is to enable cell growth in three dimensions, aggregation based on natural cellular affinities (facilitating co-culture of different cell types), and differentiation into 3-D tissue-like assemblies.^4^ Cells are grown on porous extracellular matrix (ECM)-coated microcarrier beads which serve as scaffolds upon which they adhere, thereby allowing cells to respond to chemical and molecular gradients in three dimensions (apical, basal, and lateral) akin to the in vivo scenario. When the RWV is completely filled with medium and rotation initiated, cells are maintained in a physiological low fluid shear suspension culture environment with high mass transfer of nutrients and waste, which is optimal for growth and differentiation.^4, 21^

We previously used the RWV to establish highly differentiated 3-D intestinal organotypic models derived from human epithelial cell lines and demonstrated their ability to predict in vivo infection outcomes and *Salmonella* pathogenic mechanisms.^7, 16–18^ While initiated from a single cell type, these epithelial models spontaneously differentiated into multiple epithelial cell types normally found in the intestine, including enterocytes, goblet cells, Paneth cells, and M/M-like cells, while exhibiting extensive tight junction formation, apical-basolateral polarity, and mucin production. Following infection with *Salmonella* Typhimurium, these 3-D epithelial models exhibited phenotypes consistent with in vivo infections in animals and humans, including alterations in tissue morphology, adherence, invasion, apoptosis, and production of cytokines.^7, 16–18^ Moreover, *S.* Typhimurium invaded 3-D intestinal cells independently of all known *Salmonella* type three secretion systems, i.e. SPI-1, SPI-2, and the flagellar secretory system,^7^ a major finding that paralleled in vivo infection observations in both animals and humans,^22–27^ and challenged the paradigm established by 2-D monolayers that SPI-1 is always required for invasion of intestinal epithelium. Similar results were recently reported using a 3-D intestinal organoid model derived from human induced pluripotent stem cells, which showed that a Typhimurium *invA* mutant was still able to invade into the model, albeit at lower levels than the wild type.^6^ Collectively, these findings demonstrate the utility of 3-D tissue models in predicting in vivo-like pathogenic mechanisms.

These results led us to postulate that our 3-D intestinal epithelial model could serve as a high fidelity platform for engineering an improved version able to better recapitulate the multicellular complexity normally encountered by *Salmonella* during enteric infection. We focused on the addition of macrophages, as they are a key immune cell type targeted by *Salmonella* during infection, and intracellular survival in these cells is essential for the virulence of this pathogen.^3, 28^ While in vivo and in vitro studies have shown that macrophages may contribute to *Salmonella*-host specificity and are able to distinguish between the closely related pathovars Typhimurium and Typhi, these studies have shown inconsistent trends, likely due to differences in experimental design and implementation.^28–31^ This area of study is of keen interest given the devastating epidemic of invasive multidrug resistant non-typhoidal *Salmonella* (iNTS) infections in sub-Saharan Africa linked to a genetically distinct sequence type (ST) of *S*. Typhimurium, ST313, which has undergone genome degradation similar to that of host-adapted *S*. Typhi.^32^ *S.* Typhimurium is classified as a generalist in terms of its host specificity, causing disease in a wide range of hosts, including humans. While usually associated with self-limiting gastroenteritis in humans, *S.* Typhimurium can cause serious systemic infection in the immunocompromised. Conversely, *S.* Typhi is a human-specific pathogen that causes systemic illness and typhoid fever.^3, 33^ Thus, there is concern that ST313 may be evolving toward a human-specific adaptation similar to Typhi. However, very limited information is available regarding host-pathogen interaction characteristics and host specificity of ST313, although recent studies using ST313 strain D23580 found that it causes invasive disease in animals and lethal disease in mice, indicating it is not a human-specific pathovar.^34–36^

Herein, we report advancement of our previously developed 3-D HT-29 intestinal epithelial model^7, 17^ into a multicellular, co-culture model that provides additional complexity relevant to the intestinal microenvironment by incorporation of immune competent cells; specifically, functional macrophages. As 3-D intestinal epithelial models have been repeatedly shown to better mimic the differentiated structure/function of intestinal tissues, and respond in a more in vivo-like manner to challenge with enteric pathogens than 2-D monolayers, our comparisons in this study thus focused on the 3-D co-culture and epithelial models.^7, 17–19, 37^ Morphological and immunohistochemical analysis of the HT-29-U937 co-culture model revealed key in vivo-like structural and functional characteristics. To assess the impact of macrophages in this new co-culture model and validate its application as a human surrogate infection platform, we applied it to characterize infection profiles between closely related *Salmonella enterica* pathovars with different disease phenotypes and host tropisms (Typhimurium strains SL1344 and ST313 D23580, and Typhi strain Ty2). To further enhance the physiological relevance of the study, bacteria were cultured under aerobic or microaerobic conditions prior to infection, as *Salmonella* must adapt to a broad range of oxygen levels during its normal life cycle both before and after entry into the host. This includes aerobic conditions prior to ingestion, anaerobic conditions in the lumen, and microaerobic conditions approaching the intestinal wall.^38^ Our studies revealed that the 3-D co-culture model could discriminate between *Salmonella* pathovars and represents an innovative model for host-specific testing that can bridge knowledge gaps between 3-D epithelial models and in vivo studies to study host-pathogen interactions and unveil infectious disease mechanisms.

To our knowledge, this work represents the first RWV-derived 3-D co-culture model of intestinal epithelium and macrophages. This study is an important step forward in developing a 3-D intestinal epithelial co-culture model that integrates immune cells to mimic the multicellular complexity of the parental tissue and its application to understand the synergistic contribution of different cell types to enteric host-pathogen interactions.

## Results

### Development of a novel 3-D co-culture model

**Fig. 2:**
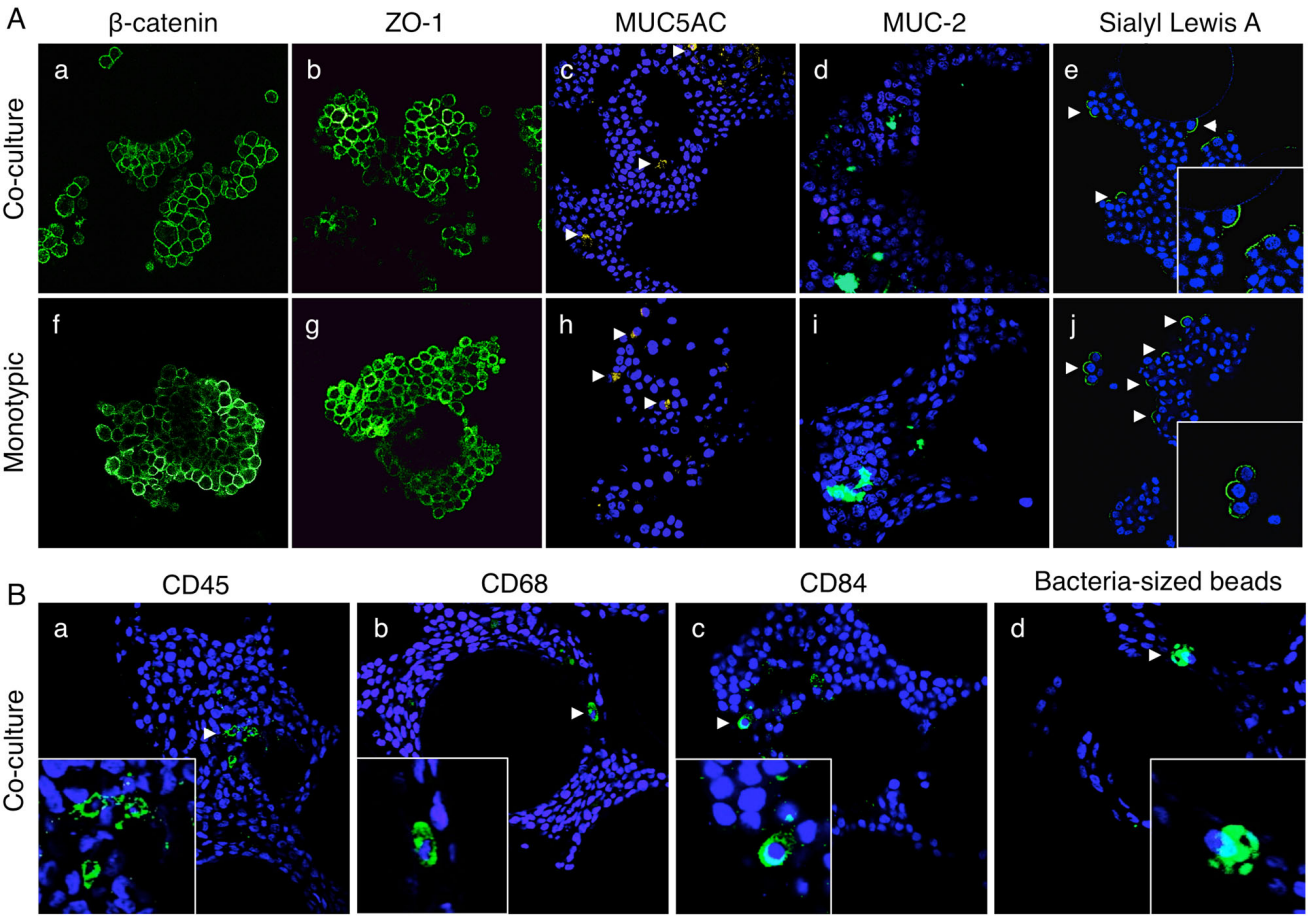
3-D co-culture model validation. **A** Adherens and tight junction markers β-catenin (**a**, **f**) and ZO-1 (**b**, **g**); mucin markers MUC5AC (**c**, **h**) and MUC2 (**d**, **i**); and M cell marker Sialyl Lewis A (**e**, **j**) shown in *green*. Cell nuclei were stained with DAPI (*blue*; **c**–**e** and **h**–**j**). **B** U937 macrophages within the 3-D co-culture model. Antibodies used to label macrophages shown in *green*: **a** anti-CD45, **b** anti-CD68, and **c** anti-CD84. Cell nuclei stained with DAPI (*blue*). **d** Internalization of bacteria-sized fluorescent beads by phagocytic U937 macrophages. The 3-D epithelial model was used as a negative control (Supplementary Fig. S1). Magnification = 400×. Arrowheads are guides for select markers

Our previous 3-D model of HT-29 colonic epithelial cells^7, 16, 17^ was advanced in this study by the inclusion of U937 monocytes that were pre-differentiated into macrophages by PMA (Figs. 1–2). The microcarrier bead scaffolds used for culturing 3-D cells resulted in establishment of an “inside-out” intestinal model, wherein the luminal side of the cells faces the culture media, which facilitates access of *Salmonella* to the host-pathogen interface naturally encountered during intestinal infection. Cells are attached to ECM-coated porous beads through their basal surface and are in contact with the external milieu at their apical surface, which is relevant to the architecture of the parental tissue. In order to develop the novel 3-D co-culture model, we had to design and optimize several experimental parameters including order of addition and ratio of the two cell types (HT-29 and U937) and the activation state of U937 cells upon addition to the model. We found that the addition of U937 cells to a partially or fully established 3-D epithelial model did not result in adequate incorporation of the U937 cells into the model, nor did it allow for the U937 cells to be incorporated beneath or within the epithelium, as would be relevant to the in vivo scenario. In addition, although we previously found that the addition of U937 monocytes to a 3-D alveolar epithelial lung model naturally stimulated their activation into functional macrophages,^10^ we did not find this to be the case when these same cells were added to our 3-D colon model in our current study. Thus, we decided to pre-bind the U937 cells to the collagen I-coated microcarrier beads and activate using PMA. Following PMA removal, the cell-bead complexes were introduced into the RWV bioreactor together with the HT-29 cells. Using this optimized procedure, we found that immunofluorescence profiling revealed the presence of differentiated intestinal epithelial cells as well as macrophages, the latter of which were integrated both between epithelial cells and underneath the epithelium (Fig. 2). We then attempted to enumerate the ratio of epithelial cells to macrophages within the final model; however, we were unable to successfully remove U937 cells from the microcarrier beads using either trypsin, collagenase or a combination of the two enzymes.

### Validation of newly developed 3-D co-culture model

Distribution of markers normally expressed by intestinal epithelium was profiled using confocal laser scanning microscopy (CLSM). Adherens and tight junctional markers β-catenin and ZO-1, respectively, were found localized at contact zones between adjacent cells in both the 3-D co-culture and epithelial models (Fig. 2A, panels a, b, f and g). Mucins (MUC5AC, MUC2) and Sialyl Lewis A antigen (M-cell marker) were expressed in the 3-D co-culture model and showed a similar expression pattern as compared to the epithelial model (Fig. 2A, panels c–e and h–j). Secretion of mucins indicates the presence of goblet cells, while expression of Sialyl Lewis A demonstrates potential presence of M cells. As with the 3-D epithelial model,^7, 17^ these findings indicate the presence of various differentiated intestinal epithelial cell types in the 3-D co-culture model.

U937 cells were identified using well-defined markers expressed by macrophages including CD45, CD68 and CD84.^9, 39–41^ Each of these markers was expressed exclusively in the 3-D co-cultures (Fig. 2B) but not in the epithelial model (Supplementary Fig. S1). Macrophages were found integrated among the epithelium of the 3-D co-culture model and were observed in the models through at least 16 days of culture (Fig. 2B). The phagocytic activity of macrophages was assessed by profiling the uptake of inert, bacteria-sized (2 µm) fluorescent beads. The 3-D co-culture, but not the epithelial model, contained cells with high concentrations of internalized fluorescent beads (Fig. 2B, panel d; Supplementary Fig. S1, panel d), indicating phagocytic activity by macrophages in 3-D co-culture.

### Application of 3-D co-culture model to study host-pathogen colonization by *Salmonella* pathovars

**Fig. 3:**
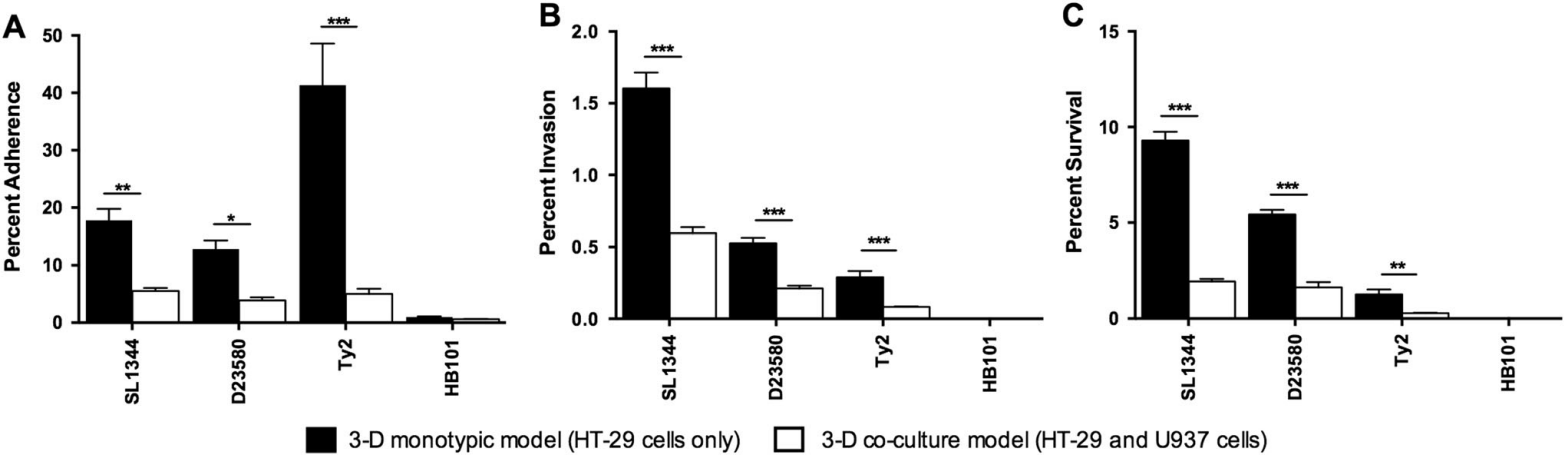
*Salmonella* pathovars colonize 3-D co-culture model at lower levels than 3-D epithelial model. Bacteria were cultured under microaerobic conditions. Triplicate wells of either the co-culture (*white bars*) or epithelial model (*black bars*) were infected at an MOI of ~10:1. Following infection, host cells were incubated for either **A** 30 min (adherence), **B** 3 h (invasion), or **C** 24 h (intracellular survival). For each time point serial dilutions of the host cell lysate were plated to assess CFU/mL. All data were normalized to the initial bacterial inoculum for each strain. Error bars represent the standard deviation from two biological replicates, each conducted in technical triplicate. Statistically significant differences for each strain between the co-culture and epithelial models are indicated as (*) *p* < 0.05; (**) *p* < 0.01; or (***) *p* < 0.001

The contribution of macrophages to *Salmonella* infection was analyzed using a gentamicin protection assay to compare colonization profiles (adherence, invasion and intracellular survival) between Typhimurium strains SL1344 and D23580, and Typhi strain Ty2 in the 3-D co-culture and epithelial models. Bacteria were cultured under both aerobic and microaerobic conditions prior to infection (Fig. 3 and Supplementary Fig. S2). All *Salmonella* strains were recovered at significantly lower numbers in the co-culture model containing functional macrophages relative to the epithelial model for all colonization time points. Decreased colonization of the 3-D co-culture model was observed regardless of the oxygen condition under which the bacteria were cultured (Fig. 3 and Supplementary Fig. S2).

### Model-specific differences in adherence profiles of Typhimurium vs. Typhi

We observed distinct patterns in the adherence of *S*. Typhi strain Ty2 relative to the two NTS strains (SL1344 and D23580). Ty2 adhered to the 3-D epithelial model at greater levels than either of the NTS strains (Fig. 3A and Supplementary Fig. S3A). This difference was completely abrogated in the 3-D co-culture model containing functional macrophages, as all strains displayed decreased adherence with no statistical differences observed (Fig. 3A and Supplementary Fig. S3A). Although all strains adhered to the 3-D co-culture model at lower levels than the epithelial model, there was a significantly larger reduction in the adherence of Ty2 compared to the NTS strains (Supplementary Fig. S3B). Regardless of oxygen conditions, Ty2 displayed an ~8-fold reduction in adherence to the co-culture model relative to the epithelial model, while NTS strains only displayed a 3 to 4-fold reduction in adherence. The large decreases in *Salmonella* adherence to the 3-D co-culture model could potentially be due to changes in the expression and/or localization of surface receptors in the host that occurred with the inclusion of activated macrophages with the epithelial cells. Previous studies have shown that the cystic fibrosis transmembrane conductance regulator (CFTR) is an important receptor for Typhi^42, 43^ and has also been found to internalize within colonic crypt epithelial cells in a mouse model of Typhimurium-induced enteritis.^44^ However, immunohistochemical profiling of our uninfected 3-D epithelial and co-culture models revealed no differences in the localization or expression of CFTR that would explain these differences in adherence (data not shown).

### Oxygen-specific differences in invasion and replication/survival profiles of *Salmonella* pathovars

**Fig. 4:**
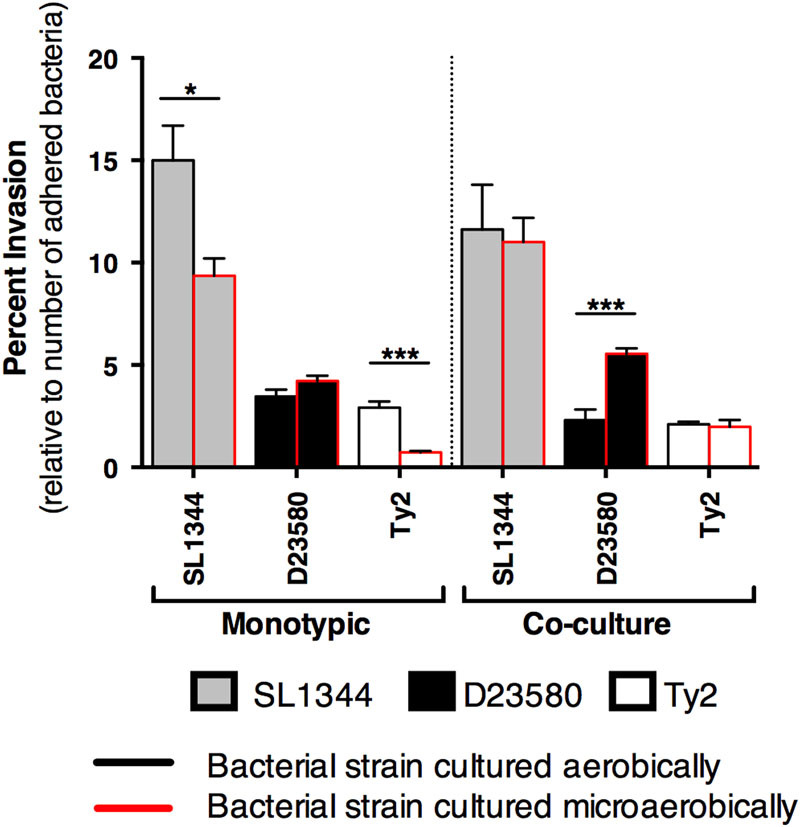
Microaerobic culture enhances the invasiveness of adherent D23580 in the 3-D co-culture model. SL1344 (*gray bars*), D23580 (*black bars*) and Ty2 (*white bars*) were cultured under either aerobic conditions (*black border*) or microaerobic conditions (*red border*) and used to infect the 3-D epithelial or 3-D co-culture model. All data for each strain were normalized to the number of bacteria that adhered. Statistically significant differences for each strain between oxygen conditions are indicated as (*) *p* < 0.05 or (***) *p* < 0.001. Error bars represent standard deviation from two biological replicates, each conducted in technical triplicate

**Fig. 5:**
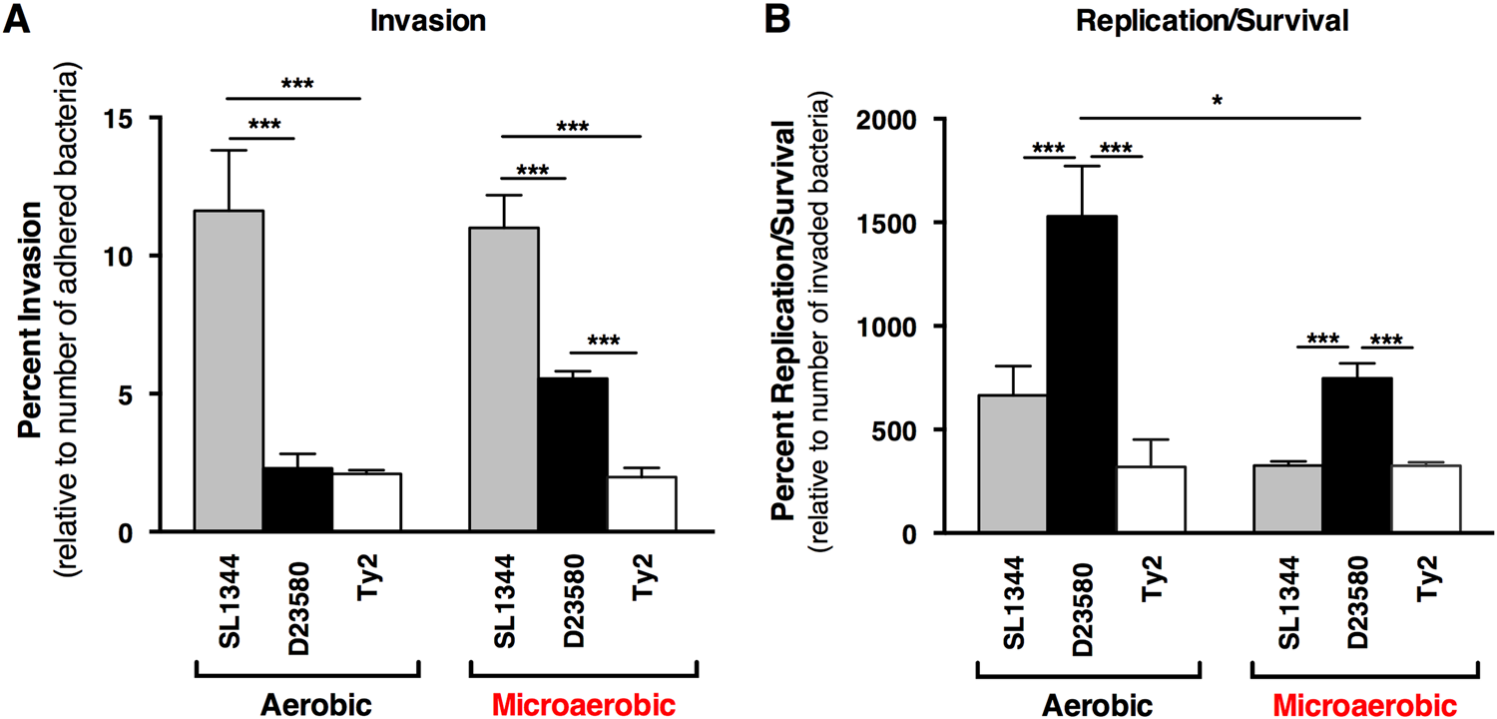
D23580 displays enhanced intracellular survival relative to SL1344 and Ty2 following invasion in the 3-D co-culture model. **A** Percent invasion relative to number of adhered bacteria. **B** Percent replication/survival relative to number of invaded bacteria.﻿﻿ Following invasion, D23580 (*black bars*) replicated/survived better than both SL1344 (*gray bars*) and Ty2 (*white bars*) (data normalized to the number of invaded bacteria). Bacteria were cultured under microaerobic or aerobic conditions. Statistically significant differences between strains are indicated as: (*) *p* < 0.05 or (***) *p* < 0.001. Error bars represent the standard deviation from two biological replicates, each conducted in technical triplicate

When analyzed as a percentage of the number of bacteria that adhered, we found that microaerobically grown *Salmonella* exhibited different invasion profiles in the 3-D co-culture as compared to the epithelial model (Figs. 4–5). Figure 4 shows statistical comparisons for each strain between oxygen conditions. Microaerobic culture decreased invasiveness of SL1344 and Ty2 in the epithelial model, while no differences were observed for D23580 between the two oxygen conditions. Conversely, in the 3-D co-culture model, SL1344 and Ty2 displayed no oxygen-related trends, while microaerobic culture enhanced the invasion of D23580. These data indicate that the multicellular complexity of the host model used to profile oxygen-dependent invasion properties is an important factor. We also found that D23580 displayed distinct colonization patterns relative to SL1344 and Ty2 (Fig. 5). Although D23580 and SL1344 adhered to both the 3-D co-culture and epithelial models at similar levels (Supplementary Fig. S2A), D23580 invaded both models at significantly lower levels (>4-fold) than SL1344 relative to the number of adhered bacteria (Fig. 5A). However, D23580 replicated/survived at higher levels in both 3-D models than either SL1344 or Ty2 following invasion (Fig. 5B and Supplementary Fig. S4). This occurred when the bacteria were cultured under either oxygen condition, with the aerobically cultured D23580 replicating/surviving approximately 2.1-fold higher than microaerobically cultured bacteria in the 3-D co-culture model (Fig. 5B, *p* < 0.05). No difference between the two oxygen conditions was observed in the replication/survival of D23580 in the 3-D epithelial model (Supplementary Fig. S4).

### Immunohistochemical profiling of 3-D models following infection

**Fig. 6:**
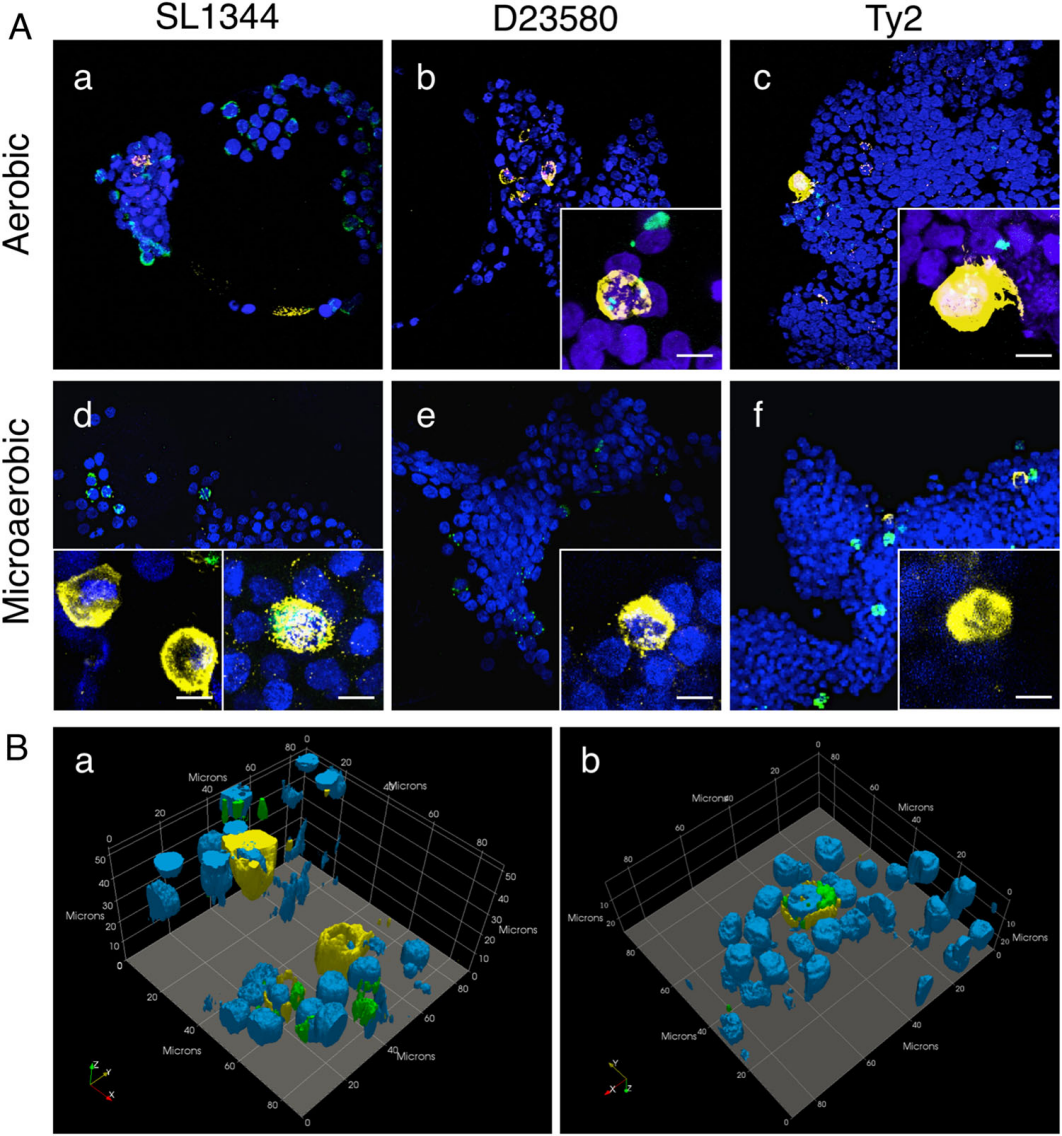
Different co-localization patterns of *Salmonella* pathovars in the 3-D co-culture model. **A** Cells were fixed 24 h.p.i. with SL1344 (**a**, **d**), D23580 (**b**, **e**) or Ty2 (**c**, **f**) that were cultured aerobically (**a**–**c**) or microaerobically (**d**–**f**). Time-matched uninfected control is shown in (Supplementary Fig. S6). Samples co-stained with CD45 antibody (*macrophages, yellow*) and anti-*Salmonella* antibody (*green*, appears white when overlaid with CD45 signal). Cell nuclei stained with DAPI (*blue*). Insets highlight representative co-localization patterns for bacteria and macrophages. When SL1344 was grown microaerobically, macrophages were either empty (**d**, *left inset*) or contained internalized bacteria (**d**, *right inset*). When D23580 and Ty2 were grown microaerobically, the majority of the macrophages were empty (insets). Magnification = 400×. Scale bar = 10 µm. **B** 3-D perspective of (**d**) insets showing **a** empty macrophages and **b** SL1344-containing macrophages. Image generated in ParaView

**Fig. 7:**
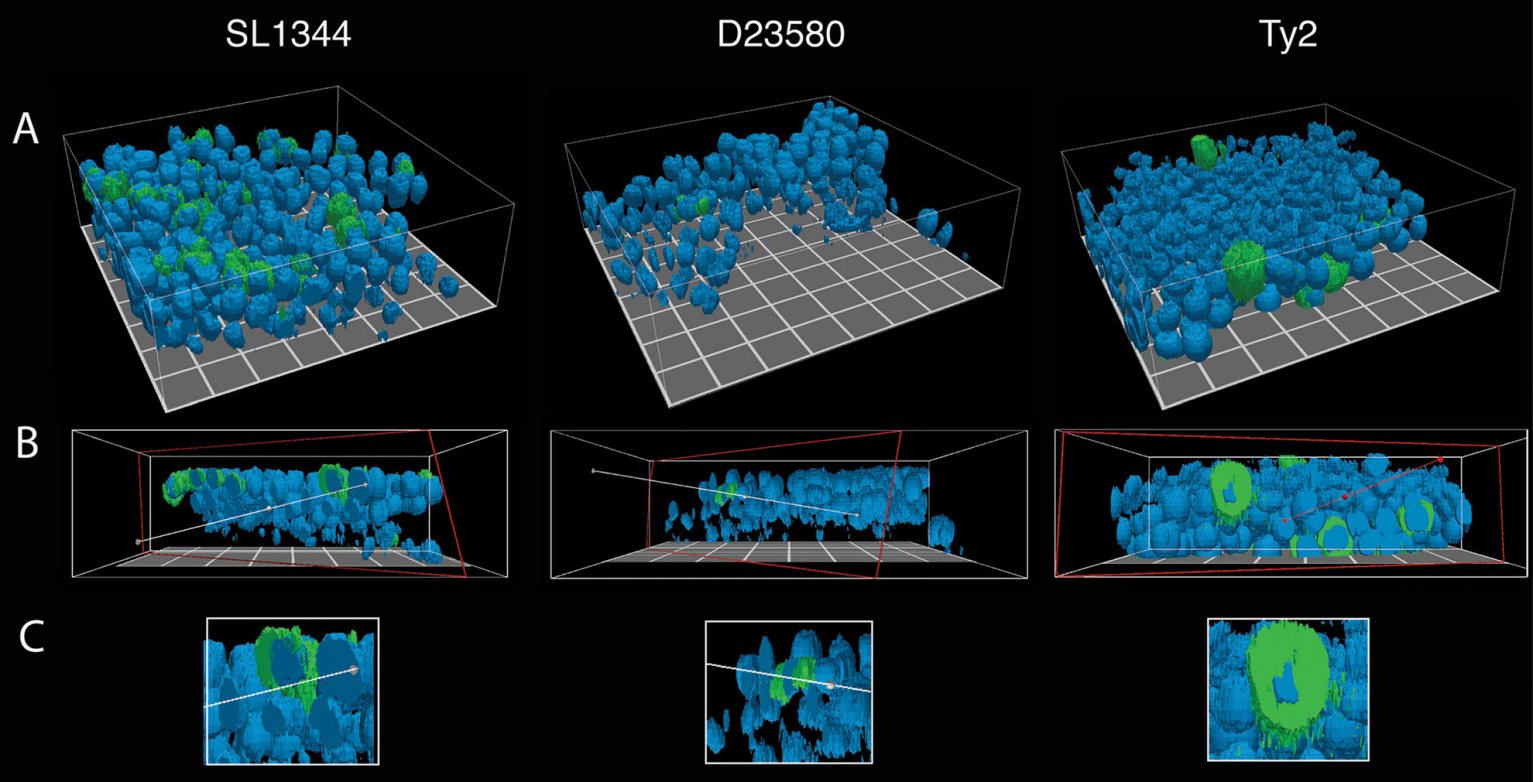
3-D perspective of intracellular *Salmonella* in epithelial cells. The co-culture model was fixed 24 h.p.i. with SL1344, D23580, or Ty2 grown microaerobically, stained with anti-*Salmonella* antibody (*green*) and DAPI (*blue*, nuclei), and imaged with CLSM (Magnification = 400×). Images were rendered as a 3-D perspective surface using an isovolume filter with ParaView (see Experimental Procedures). **A** Oblique view, **B** Axial view showing a cut-away slice, **C** digitally cropped image from (**B**) highlighting infected epithelial cells

**Fig. 8:**
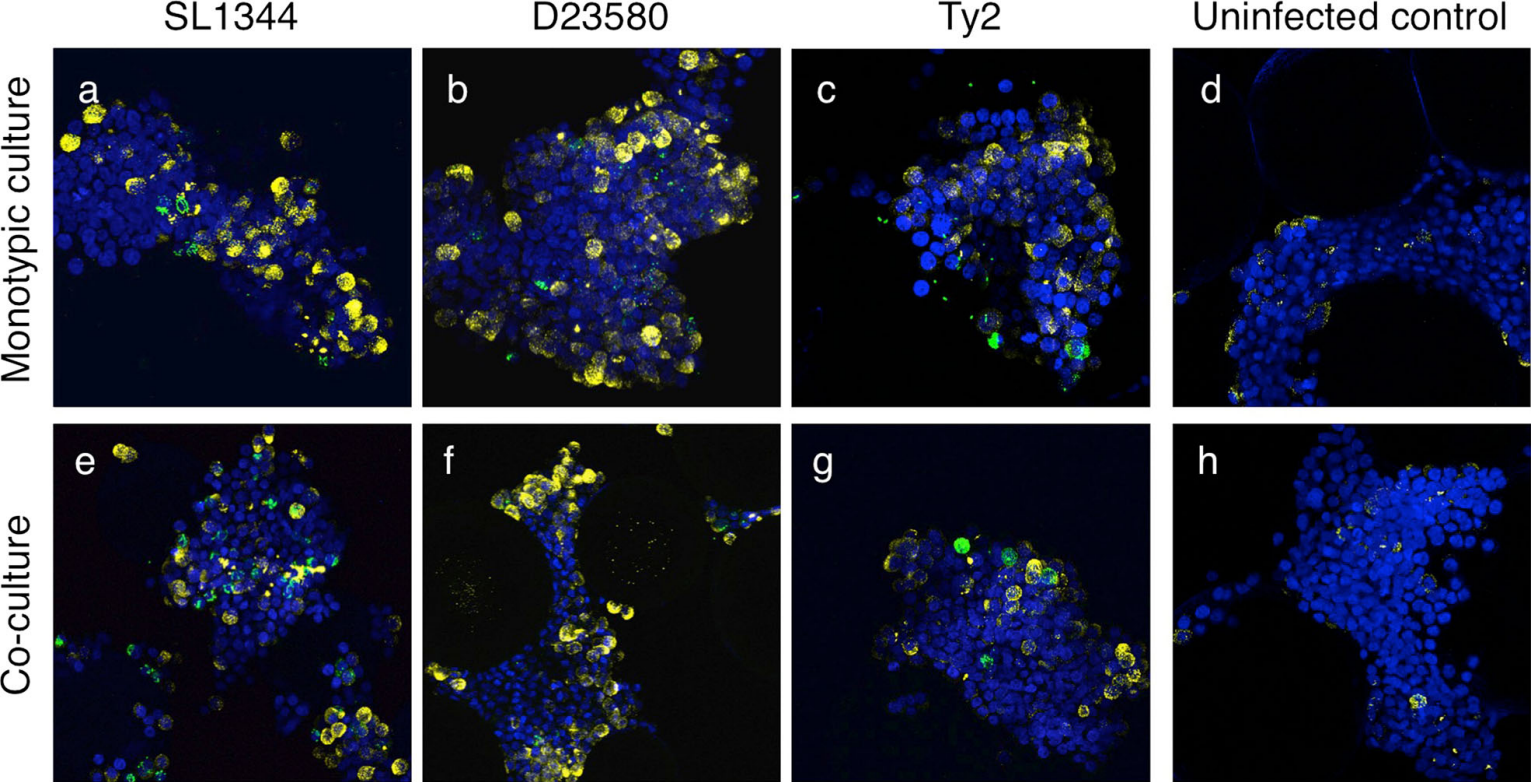
MUC5AC production by the 3-D co-culture and 3-D epithelial models following infection with *Salmonella* pathovars cultured under microaerobic conditions. The 3-D co-culture model (**a**–**d**) and 3-D epithelial model (**e**–**h**) were fixed 24 h.p.i. with either SL1344 (**a**, **e**), D23580 (**b**, **f**) or Ty2 (**c**, **g**) that had been cultured under microaerobic conditions. Time-matched uninfected controls are shown (**d**, **h**). Cultures were co-stained with a MUC5AC antibody (*yellow*), an anti-*Salmonella* antibody (*green*), and the cell nuclei stained with DAPI (*blue*). Magnification = 400×

We performed immunohistochemical profiling at 24 h post-infection (h.p.i.) to characterize the distribution/co-localization patterns of the different *Salmonella* pathovars within the 3-D co-culture and epithelial models (Figs. 6–8; Supplementary Figs. S5–S6). In both of the 3-D models infected with SL1344, bacteria were often observed to co-localize with host cells as dense intracellular clusters (Fig. 6A, panel a; Supplementary Fig. S5). When the 3-D co-culture model was infected by SL1344 grown aerobically, macrophages were rarely observed and bacteria were found predominantly in epithelial cells (Fig. 6A, panel a). In contrast, when SL1344 was grown microaerobically, a greater number of macrophages were present (Fig. 6A, panel d, insets); however, while some macrophages were found to contain internalized bacteria (Fig. 6A, panel d, right inset and Fig. 6B, panel b), most were empty (Fig. 6A, panel d, left inset and Fig. 6B, panel a), with the majority of SL1344 associated with epithelial cells.

In contrast to SL1344 infection, macrophages were readily found when D23580 was grown aerobically, with bacteria associating with both epithelial cells and macrophages in 3-D co-culture (Fig. 6A, panel b). However, when D23580 was grown microaerobically, it was rarely found in macrophages (Fig. 6A, panel e, inset). Within D23580-infected cells, the bacterial load per cell (in either macrophages or epithelial cells) appeared much lower when compared to those infected with SL1344, with bacteria forming a predominantly punctate pattern with occasional intracellular clustering (Fig. 6A, panels b and e; Supplementary Fig. S5).

In line with the colonization data, the total number of host cells (regardless of host cell type) infected with Ty2 appeared to be much lower in both 3-D models relative to SL1344 and D23580. When infected cells were found, they were often completely filled with bacteria (Fig. 6A, panels c and f; Fig. 7). In the 3-D co-culture model, we observed that when Ty2 was grown aerobically, it localized predominantly to epithelial cells, with the majority of macrophages empty. In the rare event that Ty2 co-localized with macrophages, these cells were almost always completely filled with bacteria, forming ‘giant macrophages’ (Fig. 6A, panel c, inset). In contrast, we did not find any Ty2-infected macrophages when the bacteria were grown microaerobically (Fig. 6A, panel f, inset). Instead, Ty2 formed large clusters within epithelial cells (Fig. 6A, panel f; Fig. 7).

We also examined mucus production following infection. We observed enhanced expression of MUC5AC following infection in both 3-D models relative to uninfected controls (Fig. 8 and Supplementary Fig. S7), although no clear differences in mucus production between individual pathovars was observed.

## Discussion

An improved understanding of the transition between normal intestinal homeostasis and disease caused by enteric pathogens will be facilitated by development of organotypic intestinal cell culture models that mimic the 3-D architecture and multicellular complexity present in the parental tissue in vivo. In this study, we advanced our previously characterized 3-D model of human colonic epithelium^7, 16, 17^ towards a more immunocompetent 3-D model through the co-culture of HT-29 cells and phagocytic U937 macrophages using the RWV bioreactor. To our knowledge, this model represents the first RWV-derived 3-D co-culture model comprised of intestinal epithelial cells and macrophages.

The newly developed 3-D co-culture model shares key characteristics with the 3-D epithelial model from which it was derived,^17^ including apical-basolateral polarity, well-organized tight junctions, expression of mucins, and evidence of multiple epithelial cell types present in vivo, including goblet cells and M-cells (Fig. 2A). The key advancement made in this work was the incorporation of phagocytic macrophages into the epithelial model in order to more closely approximate in vivo multicellular complexity, architecture and function. This is an important feature, as the gastrointestinal mucosa contains the largest population of tissue macrophages in the body, which play a critical role in protecting the host from pathogens, including *Salmonella*.^45, 46^ A previous study reported the incorporation of lymphocytes into a 3-D RWV intestinal epithelial model for enteric infection; however macrophages were not included in the model.^47^

When we applied both our 3-D co-culture and epithelial models to evaluate the colonization profiles of distinct *Salmonella* pathovars, we found that the presence of macrophages in the 3-D co-culture model had an inhibitory effect on the adherence, invasion and survival of all strains evaluated relative to the epithelial model, which contained epithelial cells only (Fig. 3). This inhibition could be due to an overall increased resistance to colonization as a result of cross talk between the epithelial cells and the macrophages or increased killing of the bacteria by the macrophages and/or epithelial cells. In either scenario, these findings are in alignment with the known role of macrophages in vivo to protect the host against enteric pathogens^45, 48^ and further emphasize the important role of immune cells in the early interactions between the pathogen and host.

We also found that Typhi adhered to the 3-D epithelial model at much higher levels than the Typhimurium strains (Fig. 3A and Supplementary Fig. S3). This enhanced adherence was eliminated when the same strains were used to infect the 3-D co-culture model (epithelial cells plus macrophages), with the Ty2 adherence significantly more impacted by the presence of macrophages in the co-culture model than NTS strains D23580 and SL1344 (Supplementary Fig. S3, panel B). Previous monolayer studies using a single host cell type (epithelial cells or macrophages) have produced conflicting results regarding the differences in colonization between Typhi and Typhimurium, with some studies reporting that Typhimurium adheres and invades at higher levels than Typhi and others finding the precise opposite.^33, 49–51^ Genomic comparisons between Typhi and Typhimurium have revealed discrete differences between the two serovars in genes known to be important for bacterial adherence, including fimbrial and Vi capsule genes (the latter specific to Typhi).^52–57^ While some studies have characterized the phenotypic impact of those differences,^58–61^ the adherence and invasion mechanisms of Typhi are still not fully understood, due in part to the limits of the current experimental models for human-specific infectious diseases. The expression, localization and conformation of surface structures present on both the host and pathogen play important roles in initiating infection.^62, 63^ It is well established that the expression and localization of a variety of surface markers can be altered when cells are cultured in 3-D relative to their 2-D counterparts, with the former condition often closely reflecting the in vivo-scenario.^4, 10, 12, 64, 65^ Moreover, it has been previously shown in both 3-D^10^ and 2-D^66^ cultures that the co-culture of epithelial cells and macrophages can lead to alterations in the expression of cell surface proteins. Thus the large decrease we observed in *Salmonella* adherence to the 3-D co-culture model could potentially be due to changes in the expression and/or localization of surface receptors in the host that occurred with the inclusion of macrophages with the epithelial cells. It was shown previously that CFTR is an important receptor for Typhi.^42, 43^ We hypothesized that the inclusion of activated macrophages in the 3-D co-culture model could lead to altered localization and/or expression of the CFTR receptor in a manner that could explain the drastic decrease in Typhi adherence between the epithelial and co-culture models relative to the Typhimurium serovars. However, under the conditions of this study we observed no differences by immunohistochemical profiling in its localization or expression between the two models. Another alterative explanation is that the Typhi strain was killed at higher levels than the NTS strains by the either the macrophages, the epithelial cells, or both. Studies are ongoing in our laboratory to investigate the mechanistic basis for these differences.

We were particularly interested in applying our 3-D organoid models to study the colonization characteristics of ST313 strain D23580 relative to the ‘classic’ Typhimurium and Typhi strains SL1344 and Ty2, respectively. D23580, a representative ST313 clinical isolate, has undergone genome reduction similar to that of other human-restricted serovars like Typhi.^32^ ST313 strains are often referred to as ‘highly invasive’, as they are predominantly recovered from the bloodstream of patients.^32, 35, 50, 67, 68^ However, results from research into the invasiveness and survival of ST313 relative to classic *Salmonella* strains using in vitro models have been somewhat contradictory.^36, 50, 69, 70^ Animal studies by our lab and others have indicated that gastrointestinal colonization by ST313 (including D23580) is low, with rapid spread to systemic tissues.^34, 35^ In our current study, we found that D23580 invaded both the 3-D co-culture and epithelial models at significantly lower levels than SL1344 (Fig. 3B). When we evaluated the survival of each strain relative to the number of invaded bacteria, D23580 replicated/survived better than either SL1344 or Ty2 in both of the 3-D models (Fig. 5B). These findings suggest that D23580 may spread more efficiently throughout the infected host relative to classic Typhimurium due to its inherent intracellular survival properties rather than an ability to invade at higher levels. Additional possibilities to explain the enhanced recovery of D23580 at 24 h include, (1) heterogenous populations of intracellular bacteria that are in different physiological states (including viable but not culturable organisms, or populations where the majority of bacteria have reached stationary phase), and (2) host cells that have been lysed by infection, thereby precluding the recovery of released bacteria during plating for viable cfu in gentamicin assays.

The 3-D co-culture and epithelial models could also distinguish between the *Salmonella* pathovars grown under limited oxygen. As facultative anaerobes, salmonellae encounter and respond to a wide range of oxygen levels both in the environment and in vivo. Studies have reported that limiting oxygen conditions increase the adherence and invasiveness of *Salmonella* when used to infect pure monolayer cultures of either epithelial cells or macrophages.^71–73^ We did not find that low oxygen increased the adherence or invasiveness of any of the strains tested using either of our highly differentiated 3-D cell culture models when data were normalized to the initial inoculum, although Typhi did display an upward trend in adherence in the 3-D epithelial model following microaerobic culture (*p* = 0.07, Supplementary Fig. S3C).

When invasion data were analyzed relative to the number of bacteria that had adhered, we found microaerobic culture decreased invasiveness of SL1344 and Ty2, yet had no effect on ST313 strain D23580 in the epithelial model (Fig. 4). However, in the co-culture model a different trend was observed: SL1344 and Ty2 displayed no oxygen-related differences in invasion, while microaerobic culture enhanced invasion of D23580 that had adhered to the model. The enhanced invasive capabilities of D23580 pre-adapted to low oxygen was not observed in the 3-D epithelial model (the more reductionist model; Fig. 4), nor was it observed when the data were analyzed as a percentage of the initial inoculum. These results suggest that although low oxygen may not increase the ability for D23580 to adhere to the host, the bacteria that are able attach are able to invade or be taken up at higher levels than when they are pre-adapted to higher oxygen conditions. Moreover, this enhanced invasion/uptake was only observed in the 3-D co-culture model, indicating that the macrophages play a key role in this increased invasion phenotype either directly through increased uptake of D23580 or indirectly through cross talk to the epithelium. Intriguingly, although microaerobic culture enhanced the invasiveness of D23580 that had adhered, it did not enhance the survival of those that invaded as much as when the bacteria were cultured aerobically (Fig. 5B). This difference was not observed in the 3-D epithelial model for D23580 grown under the two oxygen conditions (Supplementary Fig. S4).

One advantage of using a multicellular in vitro host model system is the ability to visualize the co-localization patterns of each strain relative to the different host cell types. We found that each of the *Salmonella* pathovars displayed distinct patterns in their clustering and host cell specificity (Fig. 6). Regardless of the model being infected (3-D co-culture or epithelial), SL1344-infected cells were abundant in samples imaged at the intracellular survival/replication time point (24 h.p.i.), with the bacteria forming dense intracellular clusters within infected cells. D23580 formed more punctate patterns with occasional intracellular clustering and what appeared to be lower numbers of bacteria relative to SL1344- or Ty2-infected models. Although Ty2-infected cells were more difficult to find relative to SL1344 and D23580, when infected cells were found (epithelial cells or macrophages), they were completely packed full of bacteria. These distinct phenotypes observed may be related, in part, to differences in the survival and/or dissemination strategies between the three strains. During *S. enterica* infections, bacteria grow within cells and are subsequently re-distributed among nearby uninfected cells in order to form new infection foci that become pathological lesions.^74–77^ There is a heterogeneous expansion within tissues, with most phagocytes displaying low bacterial numbers and small number heavily infected.^74, 76–78^ It has been previously shown in mice that *Salmonella* strains defective in SPI-2 are still able to grow to high intracellular densities, but have difficulty exiting the cells and re-distributing within tissues, leading to a lower net growth within organs and higher bacterial loads for the small numbers of infected cells.^78^ This phenotype resembles what we observed with Typhi in our human 3-D organotypic models. Several SPI-2 effectors present in Typhimurium are missing (*sseI, gogB, spvB, spvC, sseK1, sseK2,* and *sseK3*) or inactivated (*sopD2* and *sseJ*) in Typhi.^52–54, 79, 80^ These differences may lead to different dissemination strategies between the serovars in human tissues; an intriguing prospect that will be investigated using these models in future studies.

We also observed that the oxygen condition under which the bacteria had been cultured prior to the start of the infection altered the host cell co-localization distribution pattern between epithelial cells and macrophages (Fig. 6 and Supplementary Fig. S5). When SL1344 was cultured aerobically, it was very difficult to locate any macrophages in the co-culture model at 24 h.p.i., suggesting that SL1344 may have induced apoptosis in these cells; a phenotype previously observed in SL1344-infected macrophages.^30, 50, 70, 81^ In contrast, macrophages were found in D23580- and Ty2-infected models, with both strains co-localizing with intestinal epithelial cells and macrophages when the bacteria were cultured aerobically. These findings are in agreement with reports showing that SL1344 induces higher levels of apoptosis in macrophages than Typhi^30, 70, 81^ and ST313 strains^50, 70^ when cultured under aerobic conditions. Interestingly, when SL1344 was cultured under low oxygen conditions, macrophages were readily found within the model, some of which contained bacteria while others were empty (Fig. 6A, panel d **)**. Likewise, we found more empty macrophages in the model when D23580 and Ty2 were cultured microaerobically (Fig. 6A, panels e and f). Possible explanations for these infection patterns following culture of the bacteria under low oxygen conditions prior to infection include: (1) pathogen avoidance of macrophages, (2) specific targeting of epithelial cells, (3) enhanced intracellular killing within macrophages, and/or (4) a masking effect whereby the macrophages are unable to detect the pathogen, leading to lower levels of uptake. When we compared our co-culture imaging results at 24 h to the co-culture colonization data at the same time point (Fig. 5B), both SL1344 and D23580 displayed an ~2-fold decrease (*p* < 0.05) in replication/survival when the bacteria were cultured under oxygen-limiting conditions relative to aerobic cultures. However, Ty2 displayed no significant differences in replication/survival between the two oxygen conditions (Fig. 5). Future studies will focus on further investigating the mechanisms underlying these differences.

## Conclusions

In summary, the 3-D co-culture model of human colonic epithelium in combination with immune cells that was engineered, validated and tested in this study represents a new tool to investigate mechanisms underlying enteric diseases with both non-infectious and infectious etiologies, including salmonellosis. Phagocytic macrophages were incorporated in the model in order to better recapitulate the multicellular complexity and structure of the parental tissue, thereby allowing for synergistic contributions of different cell types during infection to be assessed so that host-pathogen interactions can be studied in a more physiologically relevant context. In addition, we also demonstrated that this model could be used for investigations into the impact of different environmental parameters on colonization as well as co-localization patterns of pathogens with different host cell types. These findings emphasize the power of incorporating a series of related models with increasing complexity to study infectious disease mechanisms. Using this approach, we identified key microenvironmental factors (i.e., host multicellular complexity and oxygen) important for regulating pathovar-specific invasion and host co-localization properties. We continue to enhance the physiological relevance of these models by incorporation of additional cell types that are normally found in the tissue in vivo, including the use of primary cells, dendritic cells, B cells and T cells, which will continue to advance our mechanistic understanding of enteric infectious disease in the context of the host-microenvironment.

## Materials and methods

### Bacterial strains and growth conditions

*Salmonella* Typhimurium SL1344 and D23580, *S*. Typhi Ty2, and *E. coli* HB101 were used in this study. Bacterial cultures were initiated under aerobic conditions in Lennox broth (LB) overnight with aeration (180 rpm) at 37  °C. Overnight cultures were diluted 1:200 into fresh LB. For microaerobic studies, LB media was pre-flushed with a mixed gas comprised of 2% O_2_, 6% CO_2_, and 92% N_2_ and inoculations took place within a Coy chamber which was maintained using the same gas mixture. Oxygen levels in the chamber were monitored by an O_2_ sensor and remained between 2.1–2.4% for the duration of the culture. Both aerobic and microaerobic cultures were grown at 37 °C and 180 rpm to late log phase, and immediately thereafter used for infection assays. To confirm bacterial strains were at the same phase of growth, growth curves were performed under both aerobic and microaerobic conditions by plating for viable colony-forming units per mL (CFU/mL) and measuring the corresponding OD_600_ (data not shown).

### Human cell lines and culture conditions

The human colonic adenocarcinoma cell line HT-29 and the human monocytic cell line U937^82^ were obtained from ATCC (HTB-38 and CRL-1593.2, respectively). These cell lines were chosen for this study as both represent model cell types commonly used to study *Salmonella*-host interactions, and thus there is extensive published literature for comparison. Moreover, U937 cells are well suited for this study, as they can be differentiated into adherent phagocytic cells that exhibit many key morphological and functional properties of macrophages, including phagocytosis. GTSF-2 medium (Hyclone) supplemented with 10% heat-inactivated FBS (Life Technologies), 2.5 mg/L ITS (Sigma-Aldrich) and 2.25 g/L sodium bicarbonate was used for all studies, and cells were maintained at 37 °C, 10% CO_2_. HT-29 cells were initiated as monolayers and U937 cells as a suspension culture, the latter of which was maintained at the recommended cell density of 2 × 10^5^–2 × 10^6^ cells/mL. U937 cells (1 × 10^7^) were added to 0.25 g porous Cytodex-3 microcarrier beads (Sigma-Aldrich) that had been prepared according to the manufacturer’s instructions. Phorbol-12-myristate-13-acetate (PMA; Sigma-Aldrich) (10^−8^ M) was added to GTSF-2 to induce characteristics of terminally differentiated macrophages.^83^ The mix was evenly distributed into 6-well plates and incubated for 48 h at 37 °C, 10% CO_2_. Following incubation, PMA-differentiated U937 cells bound to microcarrier beads were gently rinsed with GTSF-2 media and combined with HT-29 cells (2 × 10^6^). Cultures were loaded into the RWV, which was then filled completely with culture media, and bubbles removed. Cultures were incubated statically for 15–30 min. prior to initiating rotation (20 rpm), monitored daily and small bubbles removed if observed. GTSF-2 was replenished after the first 4–5 days and then every 24 h thereafter until the harvest of cultures at 15–16 days. The 3-D HT-29 epithelial cultures^17^ served as controls and were grown in parallel with the 3-D co-culture model for all studies.

### Bead uptake assays

The phagocytic activity of macrophages was assessed as previously described,^10, 84^ based on uptake of bacteria-sized (2 µM) Fluoresbrite YG Microspheres (Polysciences). Briefly, 3-D aggregates (2 × 10^6^ cells) of either the co-culture or epithelial model were incubated with 10^8^ fluorescent beads for 30 min at 37 °C and 10% CO_2_ in 6-well plates (5 mL medium). Following incubation, the aggregates were rinsed in triplicate with cold Dulbecco’s Phosphate Buffered Saline (DPBS; Invitrogen) to remove non-phagocytosed beads and fixed using 4% paraformaldehyde (PFA). Following a 30-minute incubation, aggregates were rinsed in triplicate with DPBS and further processed for imaging with CLSM as described below.

### Infection studies

Upon reaching optimal differentiation as determined by immunohistochemical profiling, 3-D cell culture models were harvested from the RWV, assayed for viability, and seeded into multi-well plates to a final density of ~1 × 10^6^ cells/well. A gentamicin protection assay was then performed as described previously.^7, 17^ Briefly, bacteria were added at a multiplicity of infection (MOI) of approximately 10. Samples were plated for viable CFU/mL for adherence (30 min), invasion (3 h) and intracellular survival (24 h). For each time point, host cells were washed 2–3 times with HBSS, lysed with 0.1% sodium deoxycholate, and serial dilutions of the lysate plated on LB agar to assess CFU per mL. Gentamicin was added after the 30-min (50 µg/mL) and 3 h (10 µg/mL) time points in order to eliminate remaining extracellular bacteria. Percent adherence was calculated by normalizing the CFU/mL obtained at 30 min to the initial bacterial inoculum for each strain. Percent invasion and survival/replication were calculated as indicated in the figure legend, either by normalizing to the initial bacterial inoculum or to the total CFU obtained for the previous time point. All statistical comparisons were made in Graphpad using the Student’s *t*-test (*p* < 0.05). Two independent biological trials were performed for each experiment, with three technical replicates per trial. Time-matched controls were included for all studies using the non-invasive *E. coli* strain HB101 and untreated samples (latter for imaging studies).

### Immunohistochemical profiling

Fixation, staining and imaging of 3-D aggregates was performed as previously described.^7, 10^ Samples were imaged using a Leica TCS SP5 microscope. Images were processed using Leica LAS software. For 3-D perspectives, image stacks acquired by CLSM were median filtered using a (3 × 3) kernel for noise reduction and then gamma corrected (gamma 0.75) to compensate for image dynamic range. Processed image stacks for each fluorescence channel were then rendered as 3-D perspective surface using an isovolume filter with the open-source image visualization engine ParaView (Henderson A. 2007. ParaView Guide, A Parallel Visualization Application. Kitware, Inc.). Antibodies were purchased from Abcam (CD45, β-catenin, CD84); Life Technologies (ZO-1, MUC5AC); BioLegend (CD68); Kamiya Biomedical (Sialyl Lewis A); and KPL (*Salmonella* CSA-1). All antibodies were used at a final dilution of 1:50 in blocking solution (3% BSA, 0.05% Triton X-100 in DPBS) with the exception of anti-β-catenin and anti-Sialyl Lewis A, which were used at a final dilution of 1:100. Goat anti-mouse secondary antibody conjugated to Alexa Fluor 488 (Invitrogen) was used at a 1:500 dilution in blocking solution for detecting primary antibodies, with the exception of anti-CD68 and anti-*Salmonella* CSA-1, which were FITC-conjugated. For dual labeling purposes with CD45 and MUC5AC, a goat anti-mouse secondary antibody was used that was conjugated with Alexa Fluor 555 (Life Technologies).

## Electronic supplementary material

Supplementary Figure S1

Supplementary Figure S2

Supplementary Figure S3

Supplementary Figure S4

Supplementary Figure S5

Supplementary Figure S6

Supplementary Figure S7
