# Supplementary figures and images for: Three-dimensional organotypic co-culture model of intestinal epithelial cells and macrophages to study Salmonella enterica colonization patterns
Source: NPJ Microgravity. 2017 Feb 28;3:10. doi: 10.1038/s41526-017-0011-2 (PMC5460263; doi:10.1038/s41526-017-0011-2)

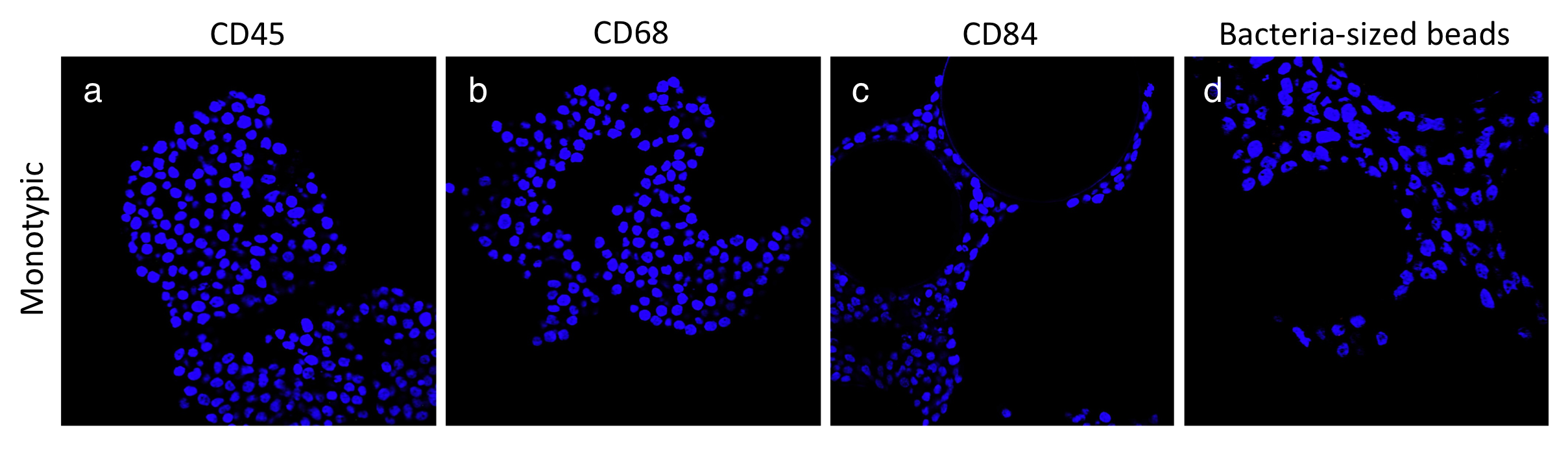

Supplement: Supplementary file 1 — Supplementary Figure S1 [file 41526_2017_11_MOESM1_ESM.tif]

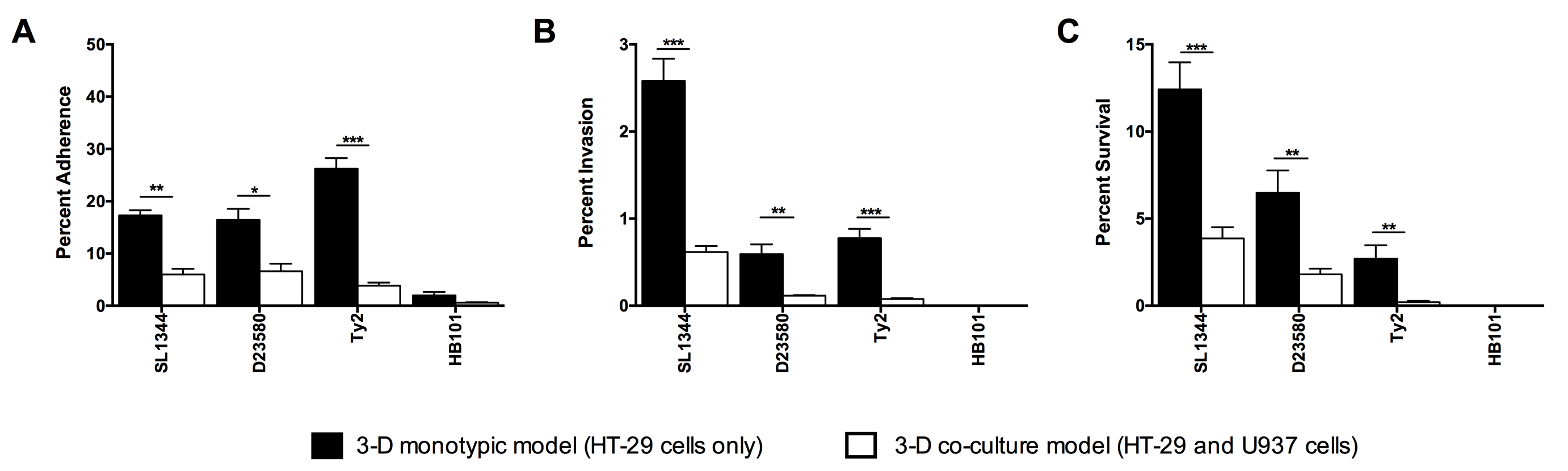

Supplement: Supplementary file 2 — Supplementary Figure S2 [file 41526_2017_11_MOESM2_ESM.tif]

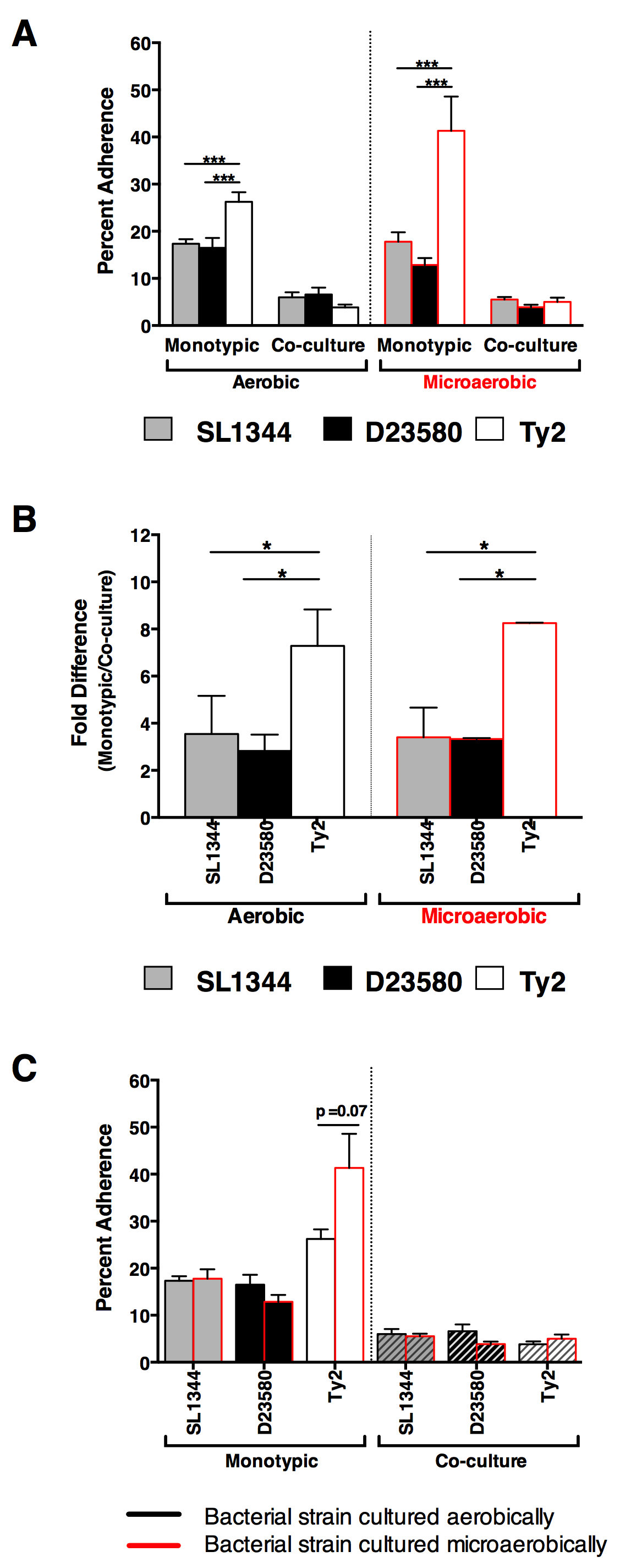

Supplement: Supplementary file 3 — Supplementary Figure S3 [file 41526_2017_11_MOESM3_ESM.tif]

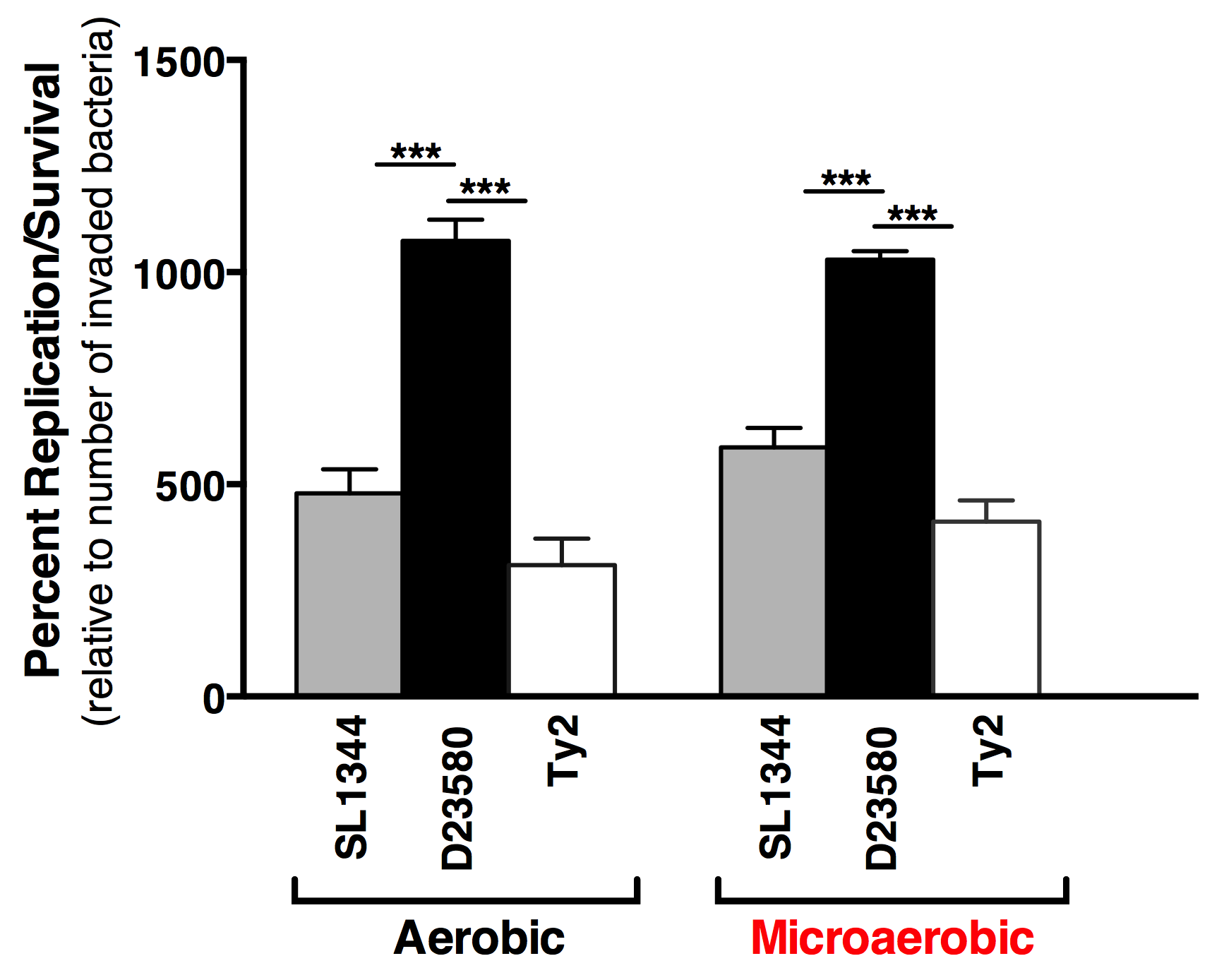

Supplement: Supplementary file 4 — Supplementary Figure S4 [file 41526_2017_11_MOESM4_ESM.tif]

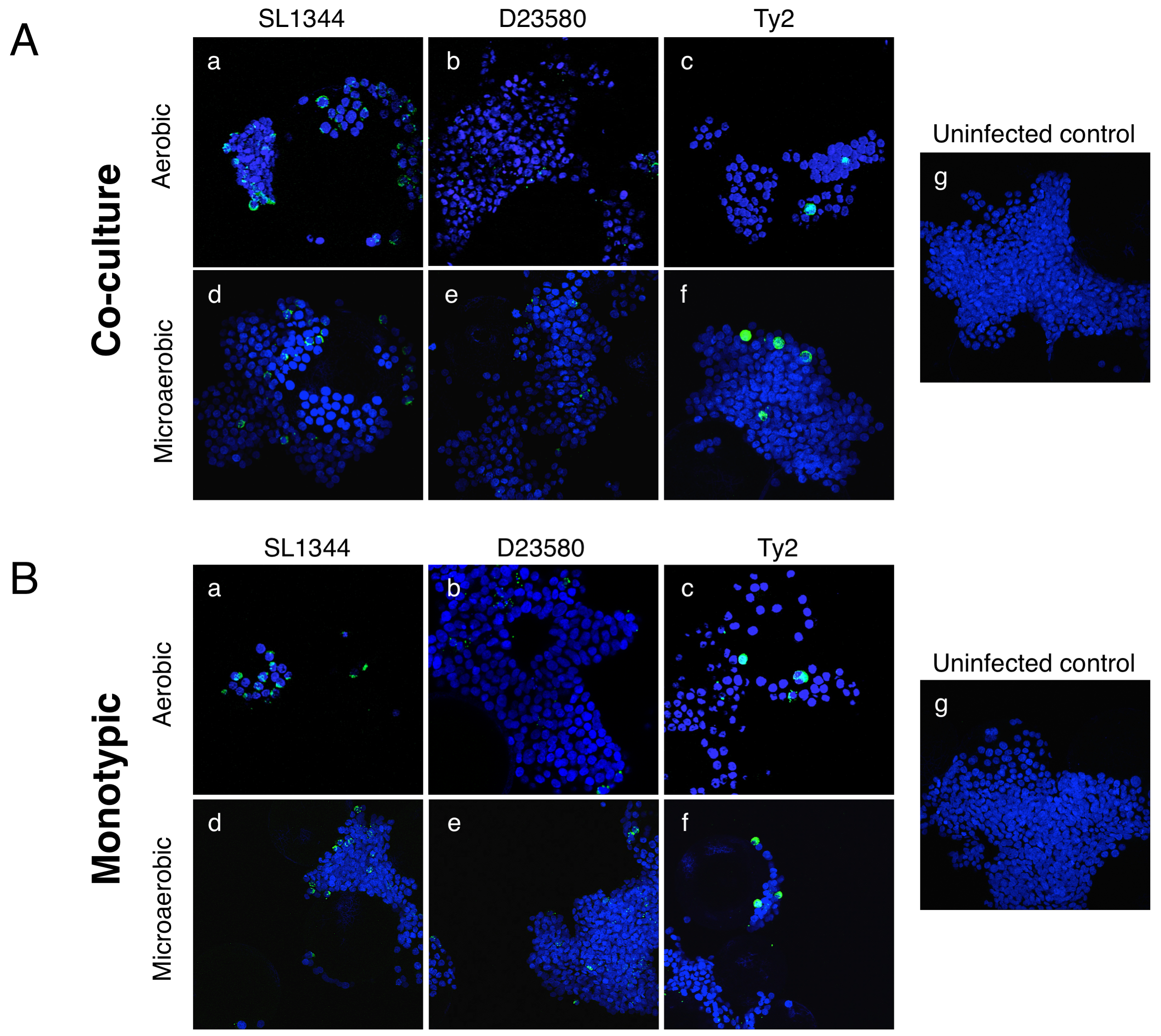

Supplement: Supplementary file 5 — Supplementary Figure S5 [file 41526_2017_11_MOESM5_ESM.tif]

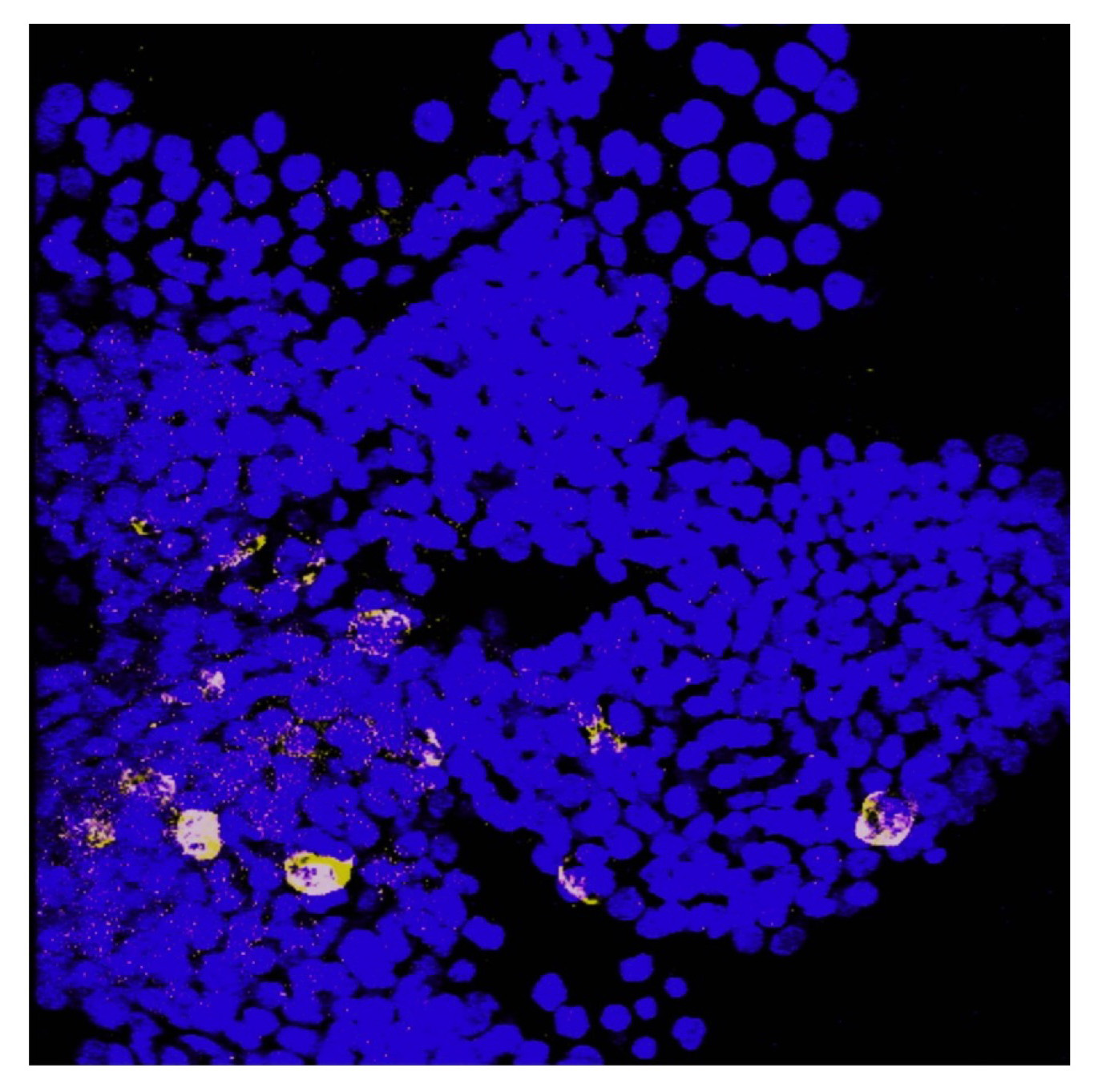

Supplement: Supplementary file 6 — Supplementary Figure S6 [file 41526_2017_11_MOESM6_ESM.tif]

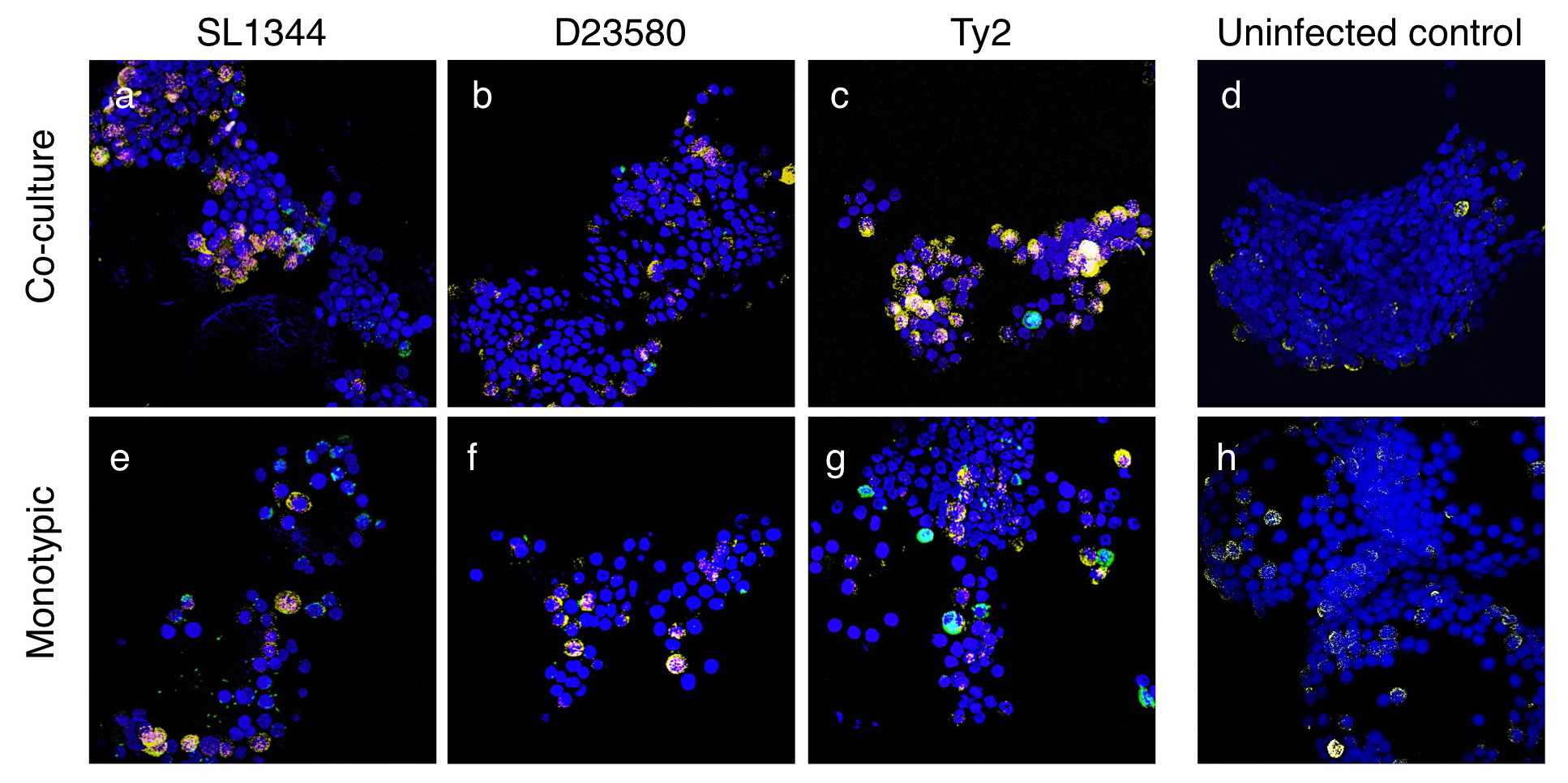

Supplement: Supplementary file 7 — Supplementary Figure S7 [file 41526_2017_11_MOESM7_ESM.tif]
